# Speckle-tracking echocardiographic abnormalities in chronic obstructive pulmonary disease: a systematic review and meta-analysis

**DOI:** 10.1186/s44348-025-00046-5

**Published:** 2025-05-08

**Authors:** Ranjini N.V., Sunil Kumar S., Nagaraj Desai, Mahesh P.A., Chaithra N., Sri Harsha Chalasani, Nikita Pal, Syed Abdul Hafeez, Chaya S.K.

**Affiliations:** 1 Department of Cardiology, JSS Medical College and Hospital, JSS Academy of Higher Education and Research, Mysore, India; 2 Namana Medical Centre, Bengaluru, India; 3 Department of Respiratory Medicine, JSS Medical College and Hospital, JSS Academy of Higher Education and Research, Mysore, India; 4 Division of Medical Statistics, School of Life Sciences, JSS Academy of Higher Education and Research, Mysore, India; 5 Department of Pharmacy Practice, JSS College of Pharmacy, JSS Academy of Higher Education and Research, Mysore, India

**Keywords:** Speckle-tracking echocardiography, Chronic obstructive pulmonary disease, Global longitudinal strain, Right ventricular free wall strain

## Abstract

Chronic obstructive pulmonary disease (COPD) is known to be associated with cardiovascular disease due to shared risk factors and its impact on the cardiopulmonary vasculature. Speckle-tracking echocardiography (STE) is an effective tool for identifying subclinical, COPD-associated right ventricular (RV) and left ventricular (LV) dysfunction before conventional echocardiography can detect it. A systematic review and meta-analysis of the literature on STE in COPD are presented. A systematic search was conducted of PubMed, Scopus, Cochrane Library, and Science Direct for papers published between 2011 and March 2023, and bias was assessed using the STROBE tool. Eleven studies were included in the two-stage meta-analysis: first among 742 COPD cases, and then a case–control design with 507 COPD cases and 259 healthy controls. Of the 11 studies analyzed, 6 were dedicated to the assessment of RV strain, and 5 studies examined LV global longitudinal strain (GLS). Significant heterogeneity was observed in the STE parameters of COPD patients (I^2^ = 95%; mean effect size: –17.055 for LV GLS and –19.098 for RV free wall strain; Z = –35.809; *P* < 0.001) and between COPD patients and controls (I^2^ = 92%; mean effect size, 2.100; Z = 8.433; *P* < 0.0001). LV and RV STE parameters were lower in the COPD group than in the healthy control group. The two-dimensional STE parameters correlated with disease severity metrices such as the BODE (body mass index, airflow obstruction, dyspnea, and exercise) index and Global Initiative for Chronic Obstructive Lung Disease (GOLD) classification, and they predicted mortality, hospitalization rates, and exercise tolerance in COPD patients. STE abnormalities are prevalent in COPD patients and can help identify subclinical LV and RV dysfunction. The presence of STE abnormalities helps in prognostication for COPD patients.

**Supplementary Information:**

The online version contains supplementary material available at 10.1186/s44348-025-00046-5.

## Background

Chronic obstructive pulmonary disease (COPD) is a prevalent chronic respiratory disease characterized by persistent and progressive airflow limitation, and it contributes to significant morbidity and mortality worldwide [1, 2]. Although the primary pathology of COPD lies within the respiratory system, emerging evidence suggests that the inflammatory state associated with COPD profoundly affects other organ systems. Among the significantly associated comorbidities, cardiovascular disease is a predictor of poor outcomes in COPD [3].

Cardiovascular diseases have a twofold significance in COPD because they share risk factors [4], and COPD can lead to changes in cardiac structure and function through factors such as hypoxemia, hypercapnia, pulmonary hypertension, and increased pulmonary vascular resistance, making patients prone to coronary artery disease, congestive heart failure, and cardiac arrhythmias independent of shared risk factors [5]. Systolic or diastolic heart failure frequently coexists with COPD, and the presence of ventricular dysfunction is associated with a poor prognosis [3, 6].

Subclinical left ventricular (LV) and right ventricular (RV) systolic and diastolic dysfunction has been seen in patients with COPD who do not have overt cardiovascular disease and have only mild airflow limitation, suggesting that cardiac complications might start to develop early in the progress of lung disease and remain subclinical for a long period [7].

Two-dimensional (2D) transthoracic echocardiography is a quick, noninvasive, reliable, widely available foundational imaging modality for assessing cardiac structure and function, RV filling pressure, tricuspid regurgitation, and valve function [8]. However, conventional echocardiography has limitations in assessing RV function due to its complex geometry and pulmonary hyperinflation, and it often fails to identify subclinical ventricular dysfunction. Newer adjunctive echocardiography-based techniques have emerged for detecting subclinical myocardial dysfunction.

Speckle-tracking echocardiography (STE) is one such method. It measures the deformation of ultrasound myocardial tissue interactions, known as speckles, throughout the cardiac cycle and provides regional and global contractility estimates. STE can detect subtle changes in myocardial function before overt symptoms or identifiable changes in conventional echocardiographic measurements occur. STE is widely used in cardiovascular research to understand myocardial mechanics and predict disease outcomes. [5, 9].

A literature search returned studies in which STE was used to assess LV and RV function in COPD patients and correlate STE results with conventional echocardiography parameters, COPD severity, and clinical outcomes. However, the STE findings were heterogeneous among the individual studies. This systematic review and meta-analysis was undertaken to provide high-quality evidence for STE abnormalities in COPD and their significance in clinical practice. Our aim was to study both RV and LV STE parameters, critically evaluate the methodological rigor of previous studies, and identify key knowledge gaps.

### Review methodology

This meta-analysis study protocol was registered in PROSPERO (No. CRD42022353218). The study findings are presented using the Preferred Reporting Items for Systematic Reviews and Meta-Analyses (PRISMA) recommendations [10]. The PRISMA checklist is provided in Supplementary File 1.

### Search strategy

Electronic databases (PubMed, Scopus, Cochrane Library, and Science Direct) were systematically searched to identify relevant studies. Cohort and cross-sectional studies of COPD patients that used STE to assess LV global longitudinal strain (GLS), RV GLS, and RV free wall strain (FWS) were included in this review. To search the databases, search phrases were used along with Boolean operators such as “AND,” “OR,” and “NOT” to combine or exclude search words.

**Fig. 1:**
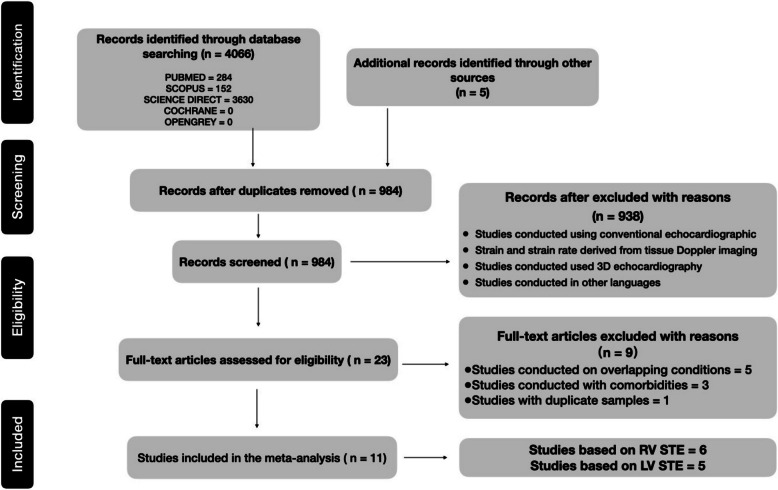
The Preferred Reporting Items for Systematic Reviews and Meta-Analyses (PRISMA) flowchart on the selection process for selecting published reports on about speckle-tracking echocardiography (STE) and chronic obstructive pulmonary disease. 3D, three-dimensional; LV, left ventricular; RV, right ventricular

The search was conducted on studies published from 2011 to March 2023. A comprehensive search was conducted using keywords such as “COPD OR Chronic obstructive pulmonary disease AND Speckle tracking echocardiography,” “COPD OR Chronic obstructive pulmonary disease AND LV GLS,” “COPD OR Chronic obstructive pulmonary disease AND RV GLS,” “COPD OR Chronic obstructive pulmonary disease AND right ventricular global longitudinal strain,” and “COPD OR Chronic obstructive pulmonary disease AND left ventricular global longitudinal strain.” Both grey literature and Google Scholar were searched in addition to the primary search. To ensure the highest quality of data, case reports, review articles, editorials, abstracts of trials without a published text, and expert comments were filtered out. Systematic screening for relevant information was conducted during the search. As depicted in Fig. 1, a structured multistep approach was followed to achieve systematic screening. Citation lists were also screened to identify additional sources that could provide further insights and information.

The citations deemed relevant in the initial screening underwent a thorough review of their titles, abstracts, and full texts to determine their relevance and suitability for this study. The first reviewer conducted a comprehensive search and shortlisted the studies that met the criteria for inclusion. A second reviewer then independently assessed the selected citations to ensure that the final selection was unbiased and based on objective criteria.

### Selection criteria

First, the electronic search results were combined, and duplicates were eliminated. Two reviewers screened all unique records by title and abstract for eligibility. Conflicts about the eligibility of a study were addressed through consensus or by referring to a third reviewer when conflicts persisted. One reviewer searched all the titles, abstracts, and appropriate full-text studies. A second reviewer conducted an independent assessment of the shortlisted citations.

Studies that measured the STE parameters in COPD patients with established cardiovascular disease or associated pulmonary diseases such as obstructive sleep apnea or interstitial lung disease and studies of patients who had undergone lung transplantation were excluded from this review. Studies published in languages other than English, those without full-text availability, and publications with duplicate samples were also excluded.

### Data extraction

During the data extraction process, a range of information was gathered: first author name, year, demographic data, study design, study population, clinical data relevant to COPD, conventional echocardiographic parameters, and STE parameters. The first reviewer extracted the data using an extraction form. A second reviewer examined the data extraction of most of the studies, which were chosen using random selection.

### Data synthesis and statistical methods

The extracted data were thoroughly analyzed using both qualitative and annotative techniques to extract the most relevant information. The extracted data were then tabulated in an extraction form to simplify the process of summarizing. This allowed for the easy identification of key findings and helped to highlight any patterns or trends within the data. The STE procedures were evaluated and compared with existing guidelines [11–13].

The studies were analyzed for speckle-tracking echocardiographic parameters, focusing on LV GLS and RV STRAIN. The strain derived by 2D-STE, particularly RV FWS, has been demonstrated to be reproducible and beneficial for clinical use. Therefore, RV FWS was used in the analysis of the results [14]. The analysis was conducted in two stages. In the first stage, all 11 studies identified in the review were analyzed. In the second stage, two studies were excluded from the analysis because they did not have a case–control design, and a further analysis was done of the remaining nine studies.

The meta-analysis used the standardized mean difference as the outcome measure. A random-effects model was fitted to the data. The amount of heterogeneity (i.e., τ^2^) was estimated using the restricted maximum-likelihood estimator [15]. In addition to the estimate of τ^2^, the Q-test for heterogeneity [16] and the I^2^ statistic are reported. When any amount of heterogeneity was detected, the prediction interval for the true outcomes is also provided. Studentized residuals and Cook's distances were used to examine whether studies might be outliers or influential in the context of the model. A regression test that used the standard error of the observed outcomes as a predictor was used to check for funnel plot asymmetry.

### Study quality

Study quality was assessed using the Strengthening the Reporting of Observational Studies in Epidemiology (STROBE) tool to identify the relevant content and methodology in each of the studies reviewed [17]. Two reviewers independently rated the research quality of each study. The scores were then compared, and any disagreements were resolved through consensus. If disagreement remained, a third reviewer was brought in to resolve it. The STROBE tool checklist is provided in Supplementary File 2.

## Results

**Fig. 2:**
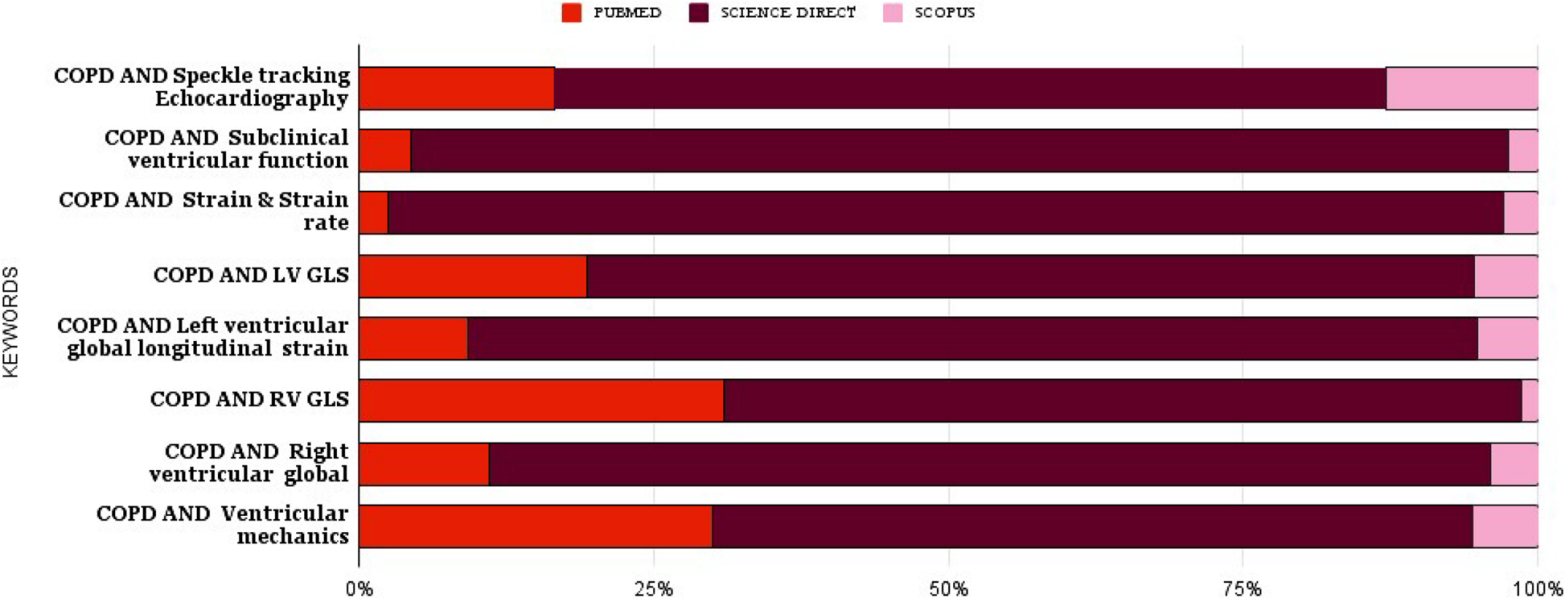
Year-wise distribution of the published studies

The original electronic database search using the keywords returned 4,066 studies, and another 5 were found using other sources. The collected data were preliminarily screened for duplicates. After the exclusion of duplicates, 938 studies underwent title and abstract screening, and 23 studies were deemed potentially eligible. After reviewing the full texts of those 23 studies, 11 studies were enrolled in this review. Figure 1 shows a flowchart of the study selection procedure. Six studies of other comorbidities, such as acute myocardial infarction, atrial fibrillation and sleep apnea; one study with duplicate samples; three studies done on the effect of interventions such as pulmonary rehabilitation; and three potential studies conducted in China, Brazil, and Indonesia that were only available in Chinese, Portuguese, and Malay, respectively, were excluded. The original electronic database search covered research studies published between 2011 and 2023. The results reveal that from 2018 to 2023, the number of studies addressing this topic increased remarkably. Figure 2. Year-wise distribution of the published studies.

This finding implies that interest in this research area has gained considerable momentum in recent years, and researchers are increasingly focusing on the subject. The upward trend in publications highlights the need for further investigation and underscores the significance of ongoing research in this field. Our primary objective of this research study was to investigate the relationship between COPD and STE by identifying relevant research studies. In our thorough search, no relevant studies were found in the Cochrane Library, and Science Direct yielded the most results.

**Fig. 3:**
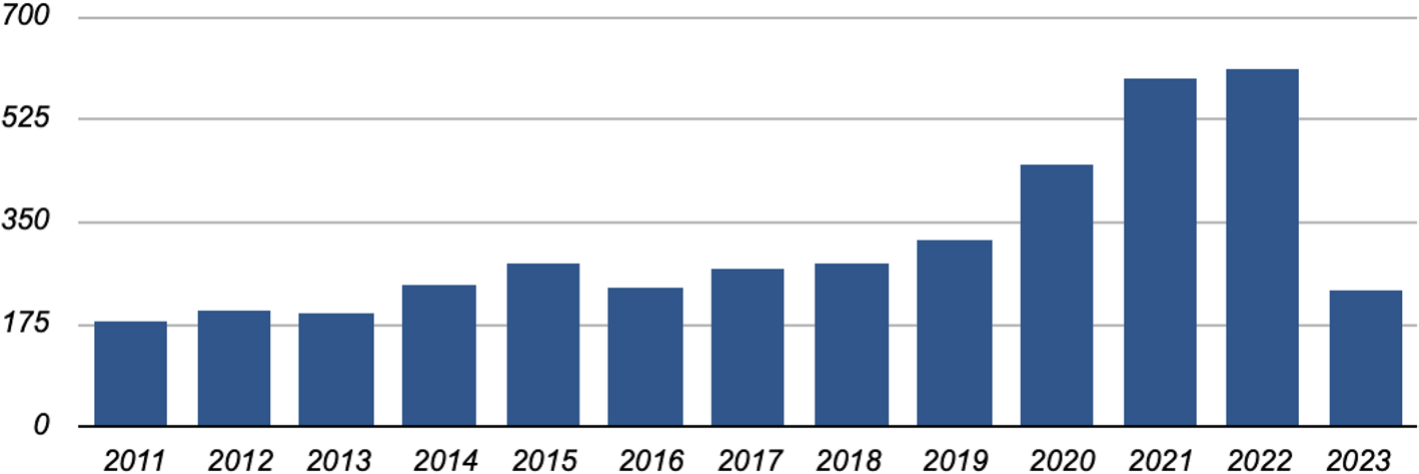
Distribution of studies based on the keywords and search engines. COPD, chronic obstructive pulmonary disease; GLS, global longitudinal strain; LV, left ventricular; RV, right ventricular

**Fig.4:**
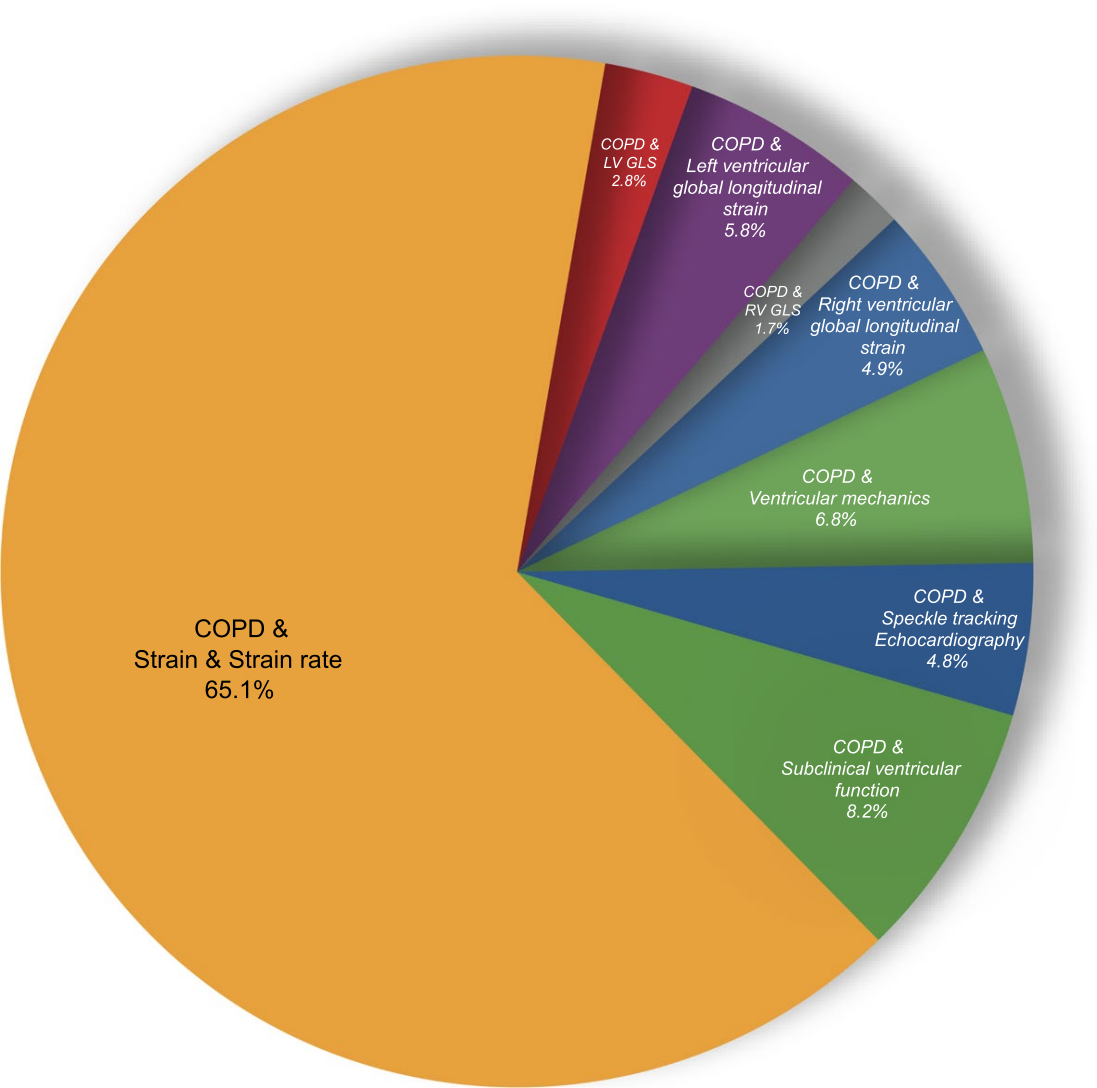
. Distribution of studies by keyword. COPD, chronic obstructive pulmonary disease; GLS, global longitudinal strain; LV, left ventricular; RV, right ventricular

The keyword “COPD AND strain AND strain rate” returned the highest number of studies, indicating a significant body of research in this area. The keyword “COPD AND RV GLS” returned the fewest studies. Figure 3 and 4 Distribution of studies based on the keywords and search engines.

The meta-analysis focused on the study populations of 11 studies [16–26]. Among the 11 studies, 9 used case–control methods, and 3 divided the samples into groups based on disease severity to determine the differences among them. The studies varied in their designs, with five cross-sectional studies, five prospective studies, and one retrospective study. Overall, 742 subjects were studied out of a total population of 1,042, with 552 male and 190 female subjects, representing a significant difference of 362 between the sexes. Nine of the 11 studies documented the subjects'body mass index (BMI). The research studies included in this meta-analysis were conducted in the following countries: the United States and Denmark (one study each), Brazil and Egypt (two studies each), and Türkiye (five studies). Within the 11 studies, the average subject age was 64.20 ± 7.15 years. Additionally, the average BMI among the subjects was 26.3 ± 5 kg/m^2^. The average 6-min walk distance (6MWD) was 330.42 ± 84.56 m. The average number of smoking packs per year was 57.67 ± 20.95 packs, indicating a relatively high level of tobacco consumption among the subjects. No significant data were provided about the duration of smoking. Of the 11 studies analyzed, 6 assessed RV strain (FWS and GLS), and 5 studies examined LV GLS. In total, the analysis included 347 subjects with LV GLS data and 395 subjects with RV FWS data. Among the 347 subjects with LV GLS results, 273 (78.7%) were male, and 74 (21.3%) were female. Among the 395 subjects with RV FWS results, 279 (70.6%) were male, and 116 (29.4%) were female. The mean predicted FEV1 was 43.45% ± 16.35%, and the mean FEV1 to FEC ratio was 56.38. Four of the 11 studies provided data on the mMRC scale, and the average mMRC score was 1.99 ± 0.79. The meta-analysis showed an average LV ejection fraction (EF) of 61.1% ± 5.16%. The tricuspid annular plane systolic excursion (TAPSE) ranged from 1.29 to 2.7 cm, with an average value of 1.95 ± 0.22 cm. The average pulmonary artery systolic pressure (PASP) was 36.29 ± 10.77 mmHg. The average RV FWS was –18.59 ± 4.07, and the average RV GLS was –17.48 ± 3.65. The average LV GLS from the five studies that reported it was –17.43 ± 3.03.

### Study characteristics

The study characteristics are presented in Table 1 [18–28]. The characteristics of the study participants are summarized in Table 2 [18–28].

**Table 1:** Characteristics of the included studies

Study	Country	Study design	Sample size			Sex		No. of smoking packs/yr	Quality assessment score
			No. of subjects	No. of cases	No. of controls	Male	Female		
Schoos et al. [22] (2013)	Denmark	Prospective study	101	90	NA	36	54	NA	19
Gökdeniz et al. [21] (2014)	Türkiye	Cross-sectional study	135	135	37	125	10	56.2 ± 25.4	17
Kalaycıoğlu et al. [25] (2015)	Türkiye	Cross-sectional study	125	125	30	115	10	52	19
Rice et al. [28] (2016)	USA	Retrospective study	54	54	NA	30	24	NA	16
Al Abbady et al. [20] (2018)	Egypt	Prospective study	50	30	20	26	4	68.80 ± 27	18
Kanar et al. [26] (2018)	Türkiye	Prospective study	69	49	41	35	14	NA	18
Kanar et al. [27] (2018)	Türkiye	Prospective study	57	46	32	28	18	NA	19
Fahim et al. [18] (2020)	Egypt	Cross-sectional study	50	50	50	44	6	59.89 ± 31.4	18
Masson Silva et al. [24] (2021)	Brazil	Cross-sectional study	126	91	NA	48	43	NA	20
Cengiz Elçioğlu et al. [19] (2022)	Türkiye	Prospective study	52	52	29	52	0	NA	18
Botelho et al. [23] (2022)	Brazil	Cross-sectional study	223	20	20	13	7	51.5 (35.8–60.0)	21

Values are presented as number only, mean ± standard deviation, or median (interquartile range)

NA, not available

**Table 2:** Clinical characteristics of the study participants

Study	Age (yr)	6MWD (m)	BMI (kg/m^2^)	FEV1 (%) predicted	FEV1/FVC ratio	LV EF	S’ RV	TAPSE (cm)	PASP	RV FWS	RV SW strain	RV GLS	LV GLS
Schoos et al. [22] (2013)	62 to 77	403 ± 113	25.75 (21 to 28.5)	52.07 (49.22 to 56.9)	49.75 (44.75 to 55)	63.9 ± 8.8	13.25 (11.75 to 14.25)	2.25 (2.12 to 2.45)	32.65 (29.37 to 38.27)	NA	NA	–23.95 (–18.9 to –30.42)	–18.4 ± 3.8
Gökdeniz et al. [21] (2014)	69.6 ± 10.6	282.75 ± 117.1	25.3 ± 4.7	47.3 ± 17.6	61.1 ± 9.9	59.1 ± 5.4	11.8 ± 3.3	2.0 ± 0.2	32.2 ± 7.5	–19.3 ± 2.2	–19.9 ± 0.8	NA	NA
Kalaycıoğlu et al. [25] (2015)	70 ± 10	NA	25 ± 4	45 ± 15	60 ± 9	59.2 ± 5.4	0.16 ± 0.03	2.3 ± 0.4	32.4 ± 7.6	NA	NA	NA	–18.58 ± 3.28
Rice et al. [28] (2016)	54 to 61	NA	NA	16 (13 to 20)	NA	65.5 (61 to 70)	NA	NA	44 (31.5 to 62)	–21.5 (–26 to –13.5)	NA	NA	NA
Al Abbady et al. [20] (2018)	62.4 ± 5.9	NA	NA	53 ± 17	54.9 ± 6	60.8 ± 4.6	NA	2.0 ± 0.2	37.9 ± 9.6	NA	NA	NA	–17.38 ± 1.3
Kanar et al. [26] (2018)	61.2 ± 10.1	337 ± 42	31.2 ± 9.3	13.4 ± 4.2	68%	58.3 ± 5.1	NA	1.66 ± 0.2	50.1 ± 18	–18.7 ± 3.9	–20.8 ± 2.8	NA	NA
Kanar et al. [27] (2018)	60.8 ± 10.2	326 ± 42.2	28.2 ± 8.4	NA	NA	57.7 ± 5.5	12.9 ± 2.93	1.66 ± 0.2	46.7 ± 15.4	–18.1 ± 3.4	NA	–20.4 ± 2.4	NA
Fahim et al. [18] (2020)	61.2 ± 8.5	206 ± 95.6	26.8 ± 5.0	64 ± 17.73	53.8 ± 11.22	63.7 ± 6.6	NA	1.66 ± 0.3	20.4 ± 12.1	–13.33 ± 7.6	–14.80 ± 5.4	–12.4 ± 3.6	–16.04 ± 4.1
Masson Silva et al. [24] (2021)	65.5 ± 8.9	427.8 ± 97.5	25.2 ± 5.6	54.5 ± 22.0	46.4 ± 14.5	NA	< 9.5: n = 6 (25%) > 9.5: n = 67	< 1.7: *n* = 5 (21%) > 1.7: *n* = 67	36.9 ± 17.6	–18.98 ± 4.11	NA	–15.48 ± 4.06	NA
Cengiz Elçioğlu et al. [19] (2022)	60.2 ± 6.2	NA	24.9 ± 4.12	45.8 ± 15.9	55.8 ± 10.3	60.5 ± 1.4	10.96 ± 0.88	1.97 ± 0.11	31.03 ± 5.9	NA	NA	NA	–14.76 ± 2.69
Botelho et al. [23] (2022)	68.4 ± 8.3	NA	24.4 ± 4.2	NA	NA	68.9 ± 6.4	NA	1.8 ± 3.7	43.2 ± 11.02	NA	NA	–17.2 ± 4.4	NA

Values are presented as range, mean ± standard deviation, or median (interquartile range)

6MWD, 6-min walk distance; BMI, body mass index; EF, ejection fraction; FEV1, Forced expiratory volume in the first second; FVC, Forced vital capacity; FWS, free wall strain; GLS, global longitudinal strain; LV, left ventricular; NA, not available; PASP, pulmonary artery systolic pressure; RV, right ventricular; TAPSE, tricuspid annular plane systolic excursion

### Quality assessment

In the quality assessment, all studies achieved a score of 16 points or higher (out of 22), which corresponds to the highest tercile. None of the studies was excluded based on insufficient methodological quality. The scores for each report are included in Table 1 [16–26].

### Meta-analysis of STE parameters in COPD patients

The first stage of the meta-analysis included all 11 studies about STE parameters in COPD patients. To ensure reliable and accurate results, a random-effects model was used for this analysis, and the effect size index was calculated as the mean value and found to be –18.398 with a 95% confidence interval (CI) of –19.182 to –17.614, meaning that the true mean effect size in the population could fall anywhere within this interval. The Z-value tests the null hypothesis that the mean effect size is zero. It was found to be –35.809 with *P* < 0.001. Using a criterion α of 0.050 and based on the Z-value and associated P-value, the null hypothesis of a mean effect size of zero can be rejected, and it is concluded that the mean effect size will not be exactly zero in populations comparable to those analyzed. The Q-statistic provides a test of the null hypothesis that all studies in the analysis share a common effect size. If all studies shared the same true effect size, the expected value of Q would be equal to the degrees of freedom (the number of studies – 1). The Q-value is 210.907 with 10 df and *P* < 0.001. Using a criterion α of 0.100, we can reject the null hypothesis that the true effect size is the same in all these studies. The I^2^ statistic is 95%, which indicates that 95% of the variance in the observed effects reflects variance in the true effects, rather than sampling error. The variance of the true effect sizes, τ^2^, is 2.586 in raw units, and τ, the standard deviation of true effect sizes, is 1.608 in raw units.

If we assume that the true effects are normally distributed (in raw units), we can estimate that the prediction interval is –21.925 to –14.298. The true effect size in 95% of all comparable populations falls in this interval.

**Fig. 5:**
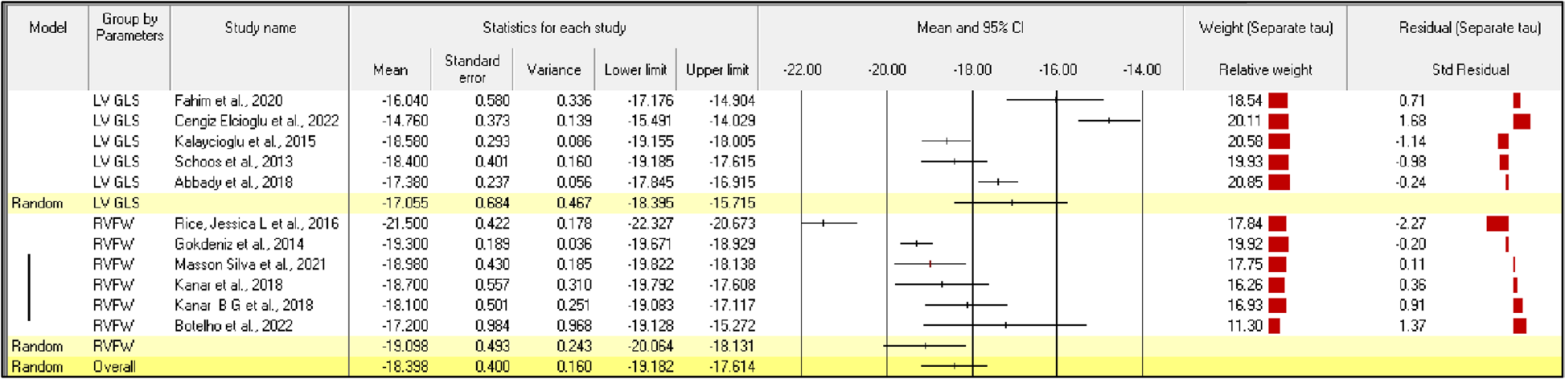
Forest plot of all studies representing speckle-tracking echocardiographic parameters in chronic obstructive pulmonary disease patients. CI, confidence interval; FWS, free wall strain; GLS, global longitudinal strain; LV, left ventricular; RV, right ventricular

In the meta-analysis of the five studies that assessed LV GLS, the mean effect size was –17.055 with a 95% CI of –18.395 to –15.715, as shown in Fig. 5. From the results, we can reject the null hypothesis that the true effect size is the same in all these studies because Z = 24.950 with *P* < 0.001 and Q = 77.747, df = 4, *P* < 0.001 with 0.100 criterion α. The meta-analysis of the six studies that assessed RV FWS revealed significant statistical heterogeneity, i.e., I^2^ = 95%, τ^2^ = 2.185, *P* < 0.0001, and the prediction interval was –22.237 to –11.872. Therefore, 95% of the variance in the observed effects reflects variance in the true effects. In the forest plot representing RV FWS in these six studies, the mean effect size was –19.098 with a 95% CI of –20.064 to –18.131, as shown in Fig. 5. From the results, we can reject the null hypothesis that the true effect size was the same in all these studies because Z = –38.722 with *P* < 0.001 and Q = 39.132, df = 5, *P* < 0.001 with 0.100 criterion α. The meta-analysis of all six studies that assessed RV GLS revealed significant statistical heterogeneity, i.e. I^2^ = 87%, τ^2^ = 1.185, P < 0.0001, and the prediction interval was –22.416 to –15.779. Therefore, 87% of the variance in observed effects reflects variance in true effects.

The results of the subgroup analysis with echocardiography variables are presented in Table 3. As shown in Fig. 5, the mean effect sizes of the studies assessing RV FWS and LV GLS differed with statistical significance (*P* < 0.0001). The average effect size for studies of LV GLS (–17.055) was significantly higher than the average effect size for studies of RV FWS (–19.098). The forest plot presented in Fig. 5 also shows the heterogeneity between studies.

**Table 3:** Random-effects model for study subjects representing speckle-tracking echocardiographic parameters in patients with chronic obstructive pulmonary disease

Group	No. of studies	Effect size		95% CI	Test of null (two-tailed)		Prediction interval	Between study		Other heterogeneity statistic			
		Standard error	Variance		Z-value	*P*-value		τ	τ^2^	Q-value	df (Q)	*P*-value	I^2^ (%)
Fixed effects analysis										77.747	4	0.000	94.855
LV	5	-	0.022	–17,615 to –17.035	–117.202	< 0.001	-	-	-	39.132	5	0.000	87.223
RV	6	-	-	–19.623 to –19.052	–132.793	< 0.001	-	-	-	116.879	9	0.000	-
Total within	-	-	0.021	-	-	-	-	-	-	94.028	1	0.000	-
Total between	-	-	-	-	-	-	-	-	-	210.907	10	0.000	95.259
Overall	11	-	0.011	–18.550 to –18.143	–176.851	< 0.001	-	-	-	-	-	-	-
Random-effects analysis													
LV	5	0.684	0.467	–18.395 to –15.715	–24.950	< 0.001	–22.237 to –11.872	1.478	2.185	-	-	-	-
RV	6	0.493	0.243	–20.064 to –18.131	–38.722	< 0.001	–22.416 to –15.779	1.089	1.185	-	-	-	-
Total between	-	-	-	-	-	-	-	-	-	5.873	1	0.015	
Overall	11	0.400	0.160	–19.182 to –17.614	–46.000	< 0.001	–22.147 to –14.650	1.608	2.586	-	-	-	-

CI, confidence interval; LV, left ventricular; RV, right ventricular

### Meta-analysis between STE parameters in COPD patients and healthy controls

The second stage of the meta-analysis was conducted in the nine studies with a case–control study design. The effect size index is the standardized difference in means (d). The overall mean effect size was 2.100 with a 95% CI of 1.612 to 2.588. The Z-value was 8.433 with *P* < 0.0001. Therefore, using a criterion α of 0.050, we reject the null hypothesis and conclude that in populations comparable to those in the analysis, the mean effect size is not precisely zero. The Q-value is 94.194 with 8 df and *P* < 0.001. Using a criterion α of 0.100, we can reject the null hypothesis that the true effect size is the same in all these studies. The I^2^ statistic is 92%, which indicates that 92% of the variance in observed effects reflects variance in the true effects rather than sampling error. The variance of true effect sizes, τ^2^, is 0.768 in d units, and τ, the standard deviation of true effect sizes, is 0.876 in d units. If we assume that the true effects are normally distributed (in d units), we can estimate that the prediction interval is –0.054 to 4.254. The true effect size in 95% of all comparable populations can be expected to fall within this interval.

**Fig. 6:**
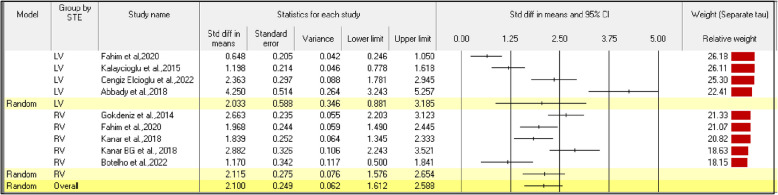
Forest plot of all studies representing speckle-tracking echocardiographic parameters in chronic obstructive pulmonary disease patients and healthy controls. CI, confidence interval; LV, left ventricular; RV, right ventricular; STE, speckle-tracking echocardiography

Five of the studies with a case–control design assessed RV FWS. The estimated average standardized mean difference based on the random-effects model was \hat{\mu} = 2.1115 (95% CI, 1.576 to 2.654). Therefore, the average outcome differed significantly from zero (Z = 7.693, *P* < 0.0001). The results of the subgroup analysis with echocardiography variables between COPD patients and control group are presented in Table 4. As shown in Fig. 6, According to the Q-test, the true outcomes appear to be heterogeneous (Q = 20.106, *P* < 0.0001, τ^2^ = 0.299, I^2^ = 80.105%). The 95% prediction interval for the true outcomes was 0.166 to 4.064. Therefore, despite some heterogeneity, the true outcomes of the studies are generally in the same direction as the estimated average outcome.

**Table 4:** Random-effects model for study subjects representing speckle-tracking echocardiographic parameters in patients with chronic obstructive pulmonary disease and healthy controls

Group	No. of studies	Effect size			95% CI	Test of null (two-tailed)		Prediction interval	Between study		Other heterogeneity statistic			
		Point estimate	Standard error	Variance		Z-value	*P*-value		τ	τ^2^	Q-value	df (Q)	*P*-value	I^2^ (%)
Fixed effect analysis														
LV	4	1.391	0.128	0.016	1.139 to 1.643	10.832	< 0.001	-	-	55.616	3	0.000	94.606	55.616
RV	5	2.148	0.121	0.015	1.912 to 2.385	17.805	< 0.001	-	-	20.106	4	0.000	80.105	20.106
Total within	-	-	-	-	-	-	-	-	-	75.722	7	0.000	-	75.722
Total between	-	-	-	-	-	-	-	-	-	18.472	1	0.000	-	18.472
Overall	9	1.793	0.008	0.008	1.621 to 1.966	20.393	< 0.001			94.194	8	0.000	91.507	94.194
Random-effects analysis														
LV	4	2.033	0.588	0.346	0.881 to 3.185	3.458	0.001	–3.450 to 7.515	1.130	1.278	-	-	-	-
RV	5	2.115	0.275	0.076	1.576 to 2.654	7.693	< 0.001	0.166 to 4.064	0.547	0.299	-	-	-	-
Total between	-	-	-	-	-	-	-	-	-	-	0.016	1	0.899	
Overall	9	2.100	0.249	0.062	1.612 to 2.588	8.433	< 0.001	–0.054 to 4.254	0.876	0.768	-	-	-	-

CI, confidence interval; LV, left ventricular; RV, right ventricular

Four studies with a case–control study design assessed LV GLS, and their mean effect size was 2.033 with a 95% CI of 0.588 to 0.346. We can reject the null hypothesis that the true effect size is the same in all the studies because Z = 3.458 with *P* = 0.001 and Q = 20.106, df = 4, *P* < 0.0001 with 0.100 criterion α. These four studies showed significant statistical heterogeneity, i.e., I^2^ = 95%, τ^2^ = 0.299, and a prediction interval of –3.450 to 7.515. Therefore, 95% of the variance in the observed effects reflects variance in the true effects.

### Methodological quality

**Fig. 7:**
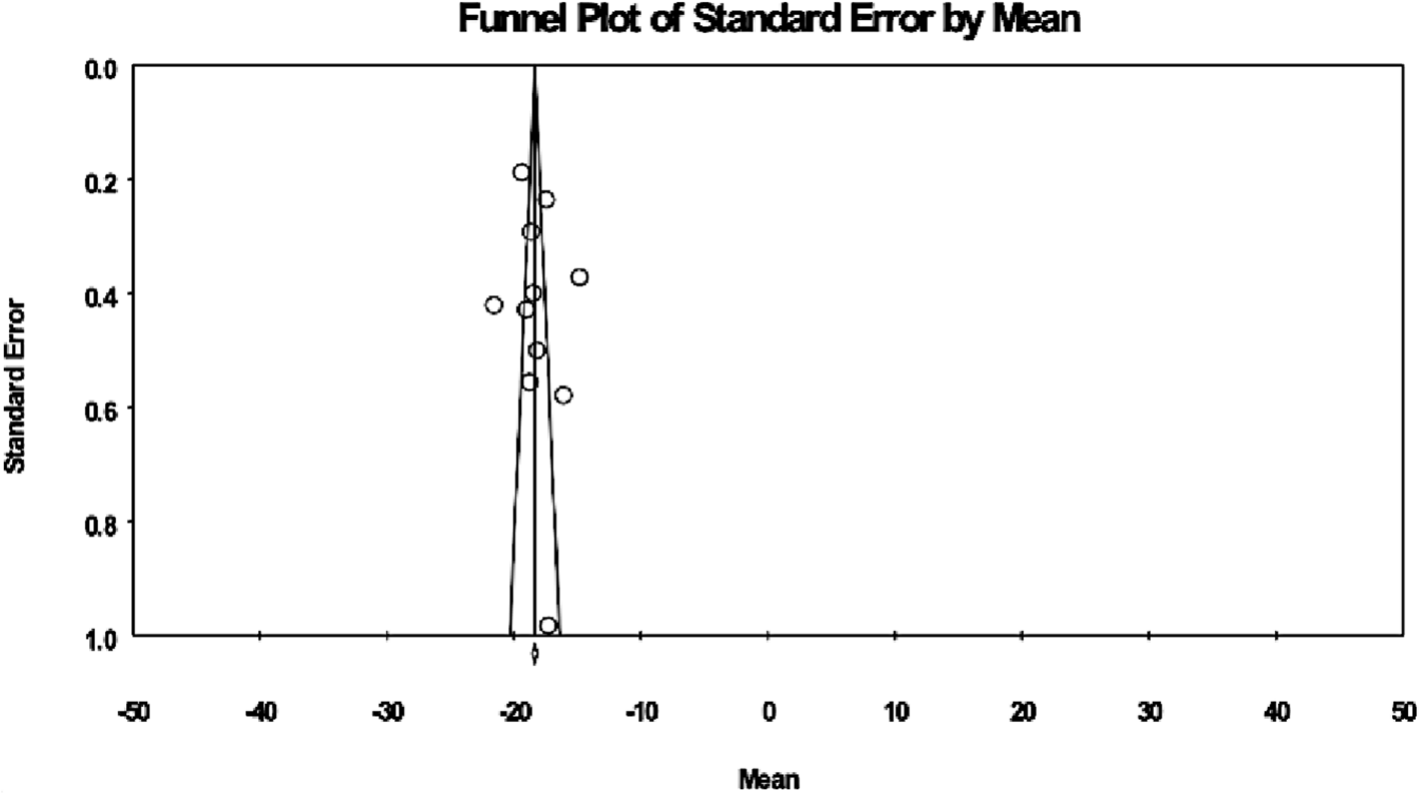
Publication bias

Publication bias was assessed using the funnel plot presented in Fig. 7. To perform Egger test, the bias dialogue was used and was carried out on Comprehensive Meta-Analysis (CMA) software and the intercept value was estimated to be 0.598 (P = 0.282). Based on the slopes of Egger test, the results suggest that no imputation was needed. Therefore, publication bias was not a concern for these studies.

## Discussion

The echocardiographic assessment of the RV and LV can influence the choice of treatment and help determine disease severity and prognosis in patients with COPD [29]. This is important because many risk factors of COPD, such as smoking and inflammatory mediators, are also associated with chronic left-sided heart failure and coronary heart disease. Additionally, pulmonary hypertension is often present in patients with COPD and can lead to left-sided diastolic dysfunction [30]. Our objective in this research was to investigate the predictive value of both RV and LV myocardial deformation, as determined using 2D-STE, in patients with COPD. 

This is the first comprehensive meta-analysis of STE abnormalities in COPD patients. Due to a lack of information on circumferential strain and radial strain in the gathered studies, we focused on longitudinal strain. The included studies used ultrasound equipment and software from various vendors to perform conventional echocardiography and analyze STE. For conventional echocardiography, five studies used a Philips ultrasound machine, three studies used a GE Healthcare ultrasound machine, one study used MyLab 30 Gold (Esaote), and two studies did not specify the manufacturer of their ultrasound equipment. Three studies each used Qlab advanced quantification software, MylabDesk Xtrain software, and Syngo Vector Velocity Imaging from Siemens Medical Solutions. Four studies used EchoPAC software (GE Healthcare), and two did not specify the software. The different software used for the strain analyses can considerably affect the results because of differences in image processing algorithms, strain tracking procedures, measurement reliability, precision, and accuracy among vendors.

We observed a notable decline in strain values shown by 2D-STE as the number of smoking pack years increased. The average 6MWD indicated moderate physical endurance and fitness among the participants. It is important to note that the variability in distance covered is relatively high among the studies, suggesting a wide range of physical abilities within the study populations. The average mMRC score from the limited available data suggests that most of the patients experienced mild to moderate dyspnea. However, it is important to note that according to the Global Initiative for Chronic Obstructive Lung Disease (GOLD) guidelines, patients with an mMRC score ≥ 2 represent a subgroup with more prominent symptoms [31]. Our findings demonstrate that studies that reported higher mMRC scores had lower 6MWD. Therefore, our findings suggest that although most of the patients experienced mild to moderate dyspnea, a significant proportion of them might have more severe symptoms. It was evident that the average EF and TAPSE values of the study participants fell within the normal range, but the strain values derived from 2D-STE were notably lower than normal. Therefore, it is crucial to closely monitor the patterns of STE parameters in COPD patients.

### RV GLS by 2D-STE

Of the 11 studies analyzed, 5 studies reported RV GLS values. The average RV GLS in COPD patients was significantly lower than in the healthy controls. The research by Fahim et al. [18] revealed similar findings, with significantly lower RV GLS in COPD patients than in the control group. That study also showed depressed RV GLS in the absence of pulmonary arterial hypertension (PAH), as assessed by tricuspid regurgitation jet velocity on echocardiography. Kanar et al. [27] revealed significantly lower RV GLS in COPD patients than in healthy controls, and they showed that RV GLS improved significantly after pulmonary rehabilitation. Chronic hypoxia and the presence of pulmonary hypertension are possible explanations for the lower RV GLS value in COPD patients because both of them can directly cause increased RV afterload and reduce RV contractility. Masson Silva et al. [24] studied echocardiographic indices for the diagnosis of RV dysfunction in COPD patients. They found that COPD patients with RV dysfunction assessed using conventional echocardiography showed lower RV GLS values. When it was measured by tissue Doppler imaging (TDI), RV GLS showed a moderate positive correlation with the tricuspid S’ value and a moderate negative correlation with the myocardial performance index (MPI). RV GLS showed a strong positive correlation with the fractional area change (FAC) and a weak correlation with TAPSE. They suggested that septal interdependence contributed by LV fiber contraction can lead to inaccuracy when diagnosing RV dysfunction using RV GLS.

On the other hand, Botelho et al. [23] studied myocardial deformation indices and three-dimensional (3D) echo parameters and concluded that RV GLS is the best predictor of RV dysfunction in patients with COPD. They compared the RV strain and strain rate measured using TDI and RV GLS measured using STE with 3D echo, but they did not include RV FWS parameters in their study.

Fahim et al. [18] reported that RV GLS decreased significantly in COPD patients as disease severity progressed. Similar findings were reported by Schoos et al. [22] in their study about the echocardiographic predictors of exercise tolerance. They found the tricuspid regurgitation jet to be the sole cardiac predictor; neither RV GLS nor LV GLS predicted exercise intolerance. The RV GLS value showed a significant drop from GOLD class I to class IV in COPD. They also found that RV GLS was negatively associated with PASP and positively associated with 6MWD. It was surprising to find that the RV GLS value correlated negatively with the LV GLS value. The study findings of Kanar et al. [27] are in agreement with the notion that RV GLS is directly associated with exercise capacity as measured by 6MWD and the BODE (BMI, airflow obstruction, dyspnea, and exercise) index. They also reported improvement in RV GLS after pulmonary rehabilitation, along with a significant improvement in PASP, the BODE index, and the mMRC scale.

These findings indicate that the early detection and assessment of impaired RV function in COPD patients can provide valuable insights into disease prognosis and management strategies. By recognizing and monitoring RV dysfunction at an early stage, clinicians could gain a better understanding of the progression and severity of COPD, enabling more effective treatment interventions and improved patient outcomes. Overall, RV GLS assessment by STE is a valuable tool for the assessment of overall RV function, the early detection of RV dysfunction, prognostication, and the guidance of therapeutic decisions.

### RV FWS by 2D-STE

Of the 11 studies analyzed, 6 studies assessed RV FWS. Our meta-analysis found a significant difference in RV FWS between the COPD patients and the healthy control group. The average RV FWS values were significantly lower in COPD patients than in the healthy control group.

Masson Silva et al. [24] markedly reduced RV FWS values in COPD patients when determining RV dysfunction using classical echocardiographic parameters. In their findings, RV FWS was the most effective myocardial deformation parameter for predicting RV dysfunction, possibly because RV FWS is not affected by septal interdependence, which makes it superior to RV GLS. That study also reported a positive correlation between RV FWS and RV FAC, S'by TDI, and TAPSE and a negative correlation between RF FWS and RV MPI. They established an RW FWS of less than –20% as an optimal cutoff suggestive of RV dysfunction.

Fahima et al. [18] found a notable difference in RV FWS between the individuals with COPD and the healthy control group. However, they revealed no correlation between RV FWS and the mean BODE index, even though significant differences in RV FWS were observed between the BODE quartiles and the control group.

Conversely, the findings of Gökdeniz et al. [21] revealed a positive correlation between RV FWS and the BODE index in individuals with COPD, along with a notable difference in RV FWS between the subjects with COPD and the healthy control group. That study divided COPD patients into two groups based on their median RV FWS value: those with RV FWS > –19.06 and those with RV FWS ≤ –19.06. They reported that patients with RVFWS ≤ –19.06, had higher disease severity, as assessed by both the BODE index and GOLD class. They also reported that the BODE index is an independent predictor of RV FWS ≤ –19.06. Additionally, the group with RV FWS ≤ –19.06 showed a significant increase in PASP.

Rice et al. [28] studied the relationships among RV FWS, resting mean PAP, and pulmonary vascular dysfunction. They found that no significant correlation between RV FWS and mean PAP, suggesting that RV strain might be a more reliable indicator of RV function than pressure. Based on the results obtained using 2D-STE, they concluded that RV FWS is a feasible and effective parameter for assessing pulmonary vascular dysfunction. They observed a strong correlation between RV FWS and elevated pulmonary vascular resistance (PVR). Specifically, patients diagnosed with PAH based on a PVR value greater than 3 Wood units (WU) exhibited significantly impaired RV FWS compared with patients without PAH, whose PVR was less than or equal to 3 WU. Furthermore, their study confirmed that the presence of PAH was associated with increased mortality and hospitalization rates, highlighting the clinical significance of these findings.

Kanar et al. [27] reported a significant difference in RV FWS between COPD patients and healthy controls. Notably, during the 3-month follow-up period after a pulmonary rehabilitation program (PRP), improvements were observed in both RV FWS and 6MWD. Those authors reported a direct relationship between RV FWS and the change in 6MWD before and after the PRP. Following the PRP, improvements were also noted in both the BODE index and MRC parameters. However, only the BODE index showed statistically significant differences. Additionally, a significant correlation was identified between RV FWS and the BODE index.

Kanar et al. [26] studied the effects of a PRP on RV dyssynchrony in COPD patients. They found that COPD exhibited both intraventricular and interventricular dyssynchrony before the PRP, with RV FW activation delayed compared with the interventricular septum and LV lateral wall in COPD patients. They also found reduced peak longitudinal systolic strain (PLSS) in COPD patients. A significant difference in RV FWS was noted before and after the PRP. The PRP was found to enhance RV FWS, improve intraventricular dyssynchrony by reducing the RV peak systolic strain dyssynchrony (PSDD) index, and improve interventricular dyssynchrony by decreasing the time-to-PLSS difference between the RV free wall and the LV lateral wall. However, even after the PRP, an intraventricular dyssynchrony was still present compared with healthy subjects. That research highlighted a time-to-PLSS difference between the RV FW and LV lateral wall > 15 ms and RV PSDD > 43 ms as independent predictors of hospitalization within 1 year. It also highlighted the presence of RV dyssynchrony, even with a normal QRS duration on electrocardiography. These findings emphasize the significant impairment of RV FWS in COPD patients, underscoring the potential role of STE as a sensitive marker for early detection of RV dysfunction in this population.

### LV GLS

Of the 11 studies analyzed, 5 studies reported LV GLS. Four of those studies were case–control studies involving COPD patients and healthy controls, and one assessed LV GLS in COPD patients and correlated it with the severity of COPD.

Our meta-analysis of the four case–control studies revealed significantly lower LV GLS values in COPD patients than in the healthy controls. However, we found heterogeneity in terms of the magnitude of LV GLS reduction and the severity of COPD. Al Abbady et al. [18], Kalaycıoğlu et al. [25], and Cengiz Elçioğlu et al. [19] reported that the mean LV GLS was lower in COPD patients and correlated with the severity of COPD. On the other hand, Fahim et al. [18] showed that LV GLS was lower in COPD patients but did not correlate with the severity of COPD, and findings from the cross-sectional study by Schoos et al. [22] showed a mean LV GLS value of –18.4 ± 3.8 in COPD patients without a clear association between LV GLS and disease severity. In that study, the mean LV GLS was higher in patients with severe COPD, but the difference was not statistically significant. The proposed explanation for increased LV GLS in those patients is as follows: (1) increased SNS activity due to hypoxia leads to increased cardiac inotropy and chronotropy, which in turn increases peak systolic strain rate; and (2) lower filling pressures are associated with a higher peak systolic strain rate, and an increase in the peak systolic strain rate increases LV GLS. In that study, LV GLS was an independent predictor of mortality due to moderate to severe COPD; however, LV GLS didn’t mediate the association between COPD severity and mortality [22, 32, 33].

Al Abbady et al. [20] reported a strong association between LV GLS and both PASP and GOLD classes. Those authors categorized the COPD cases into two subgroups based on the symptoms (mild to moderate or severe). They found significantly lower LV GLS values and higher PASP values in patients with severe COPD.

Kalaycıoğlu et al. [25] confirmed the findings of Al Abbady et al. [20]. A significant decline in LV GLS and a rise in PASP were observed as disease severity progressed from BODE quartile 1 to 4. Notably, they also stated that the BODE index quartiles were independent predictors of decreased LV GLS (≤ − 18.6). Cengiz Elçioğlu et al. [19] reported a negative correlation between LV GLS and the GOLD classification. However, they found a significant difference in LV GLS between COPD patients and the control group, even though the 3D echo-derived LV volumes did not differ significantly. They further reported that LV GLS correlated negatively with PASP and the right atrial diameter.

On the other hand, Fahima et al. [18] reported a significantly lower LV GLS in patients with COPD, compared with the control group, but found no correlation between LV GLS and disease severity as assessed by the BODE index. They noted a reduced LV GLS value in the absence of PAH.

Despite a common focus on LV GLS and COPD severity, the diverse methodologies, patient populations, and definitions of COPD severity used in the studies contribute to the observed heterogeneity. A potential rationale for this observation could be the uneven distribution of COPD cases among the various GOLD classes and BODE index categories among studies. For example, in Fahima et al. [18] and Schoos et al. [22], most of the COPD patients were classified in the moderate COPD group. Conversely, the study by Kalaycıoğlu et al. [25] included a relatively balanced distribution of COPD patients across each severity category. Also, the differences in mean PASP values among COPD patients could be a contributing factor to this heterogeneity.

Kalaycıoğlu et al. [25] divided their patients based on the median GLS values > –18.6 and ≤ –18.6 and then compared GLS with the nominal components of the BODE index. They observed that elderly patients with COPD were particularly prone to LV GLS ≤  − 18.6. Additionally, the GLS ≤  − 18.6 group exhibited higher disease severity, as assessed by GOLD classes and the BODE index. They also reported significantly higher filling pressure and PAP values in the LV GLS ≤  − 18.6 group. Cengiz Elçioğlu et al. [19] also observed a significant positive correlation between LV GLS and functional capacity, as well as exercise tolerance parameters such as the metabolic equivalents and maximum heart rate obtained during the treadmill test.

These findings provide evidence that even subtle alterations in the LV were associated with patient quality of life, even before the onset of overt dysfunction. Subclinical impairment in LV systolic function was noted without substantial LV structural alterations, as evidenced by LV and RV dysfunction in conventional echocardiography results, and some findings indicated that the dysfunction correlated with COPD severity [19].

Additionally, Schoos et al. [22] showed a negative correlation between LV GLS and BMI. A consistent decline in BMI was associated with a rise in LV GLS as disease severity progressed from GOLD class I to IV. The suggested explanation for this is the “obesity paradox” in the COPD domain. Other studies on COPD patients concluded that being overweight is a positive predictor of long-term survival [34–36]. The concept of “reverse causation,” wherein a rise in the severity or activity of COPD causes cachexia and weight loss, is one explanation for the inverse relationship between BMI and the deterioration of lung function. Increased resting energy consumption [37], nonrespiratory skeletal muscle atrophy followed by diminished peripheral oxygen availability and inactivity [38, 39], and systemic inflammation [30] are some of the hypothesized processes for that effect.

According to the results of our systematic review and meta-analysis, 2D-STE holds promise in identifying subclinical ventricular dysfunction in patients with COPD and predicting survival times. Therefore, individuals who exhibit lower strain values in 2D-STE might be candidates for further clinical evaluation and treatment to prevent the onset of heart failure.

### Limitations

1. The 2D-STE has limitations that can lead to inaccuracies, such as detecting myocardial deformation in a single plane and assuming that motion occurs strictly within that plane. Also, 2D-STE fails to track myocardial motion, especially in poor-quality images or when deformation patterns are complex. Additionally, it often focuses on global LV function, potentially overlooking localized abnormalities, so it is essential to pursue research on 3D- or 4D-STE.
  The lack of data on circumferential and radial strain hinders a thorough understanding of STE abnormalities in COPD patients. Incorporating those measurements into future research would give a more comprehensive evaluation of myocardial function, providing deeper insights into LV and RV dysfunction.
2. Hence, large case-control, follow-up studies to ascertain the effects of STE abnormalities on COPD patients.

## Conclusions

STE abnormalities are prevalent in COPD patients and correlate with conventional echocardiographic parameters in predicting RV dysfunction. STE helps to identify subclinical LV and RV dysfunction even before conventional echocardiographic abnormalities are evident. The presence of STE abnormalities correlates with a poor prognosis in COPD patients. STE is an invaluable noninvasive bedside imaging modality that can be helpful for risk stratification and prognostication in COPD patients.

## Supplementary Information

Supplementary Material 1. Preferred Reporting Items for Systematic Reviews and Meta-Analyses (PRISMA) 2020 checklist.

Supplementary Material 2. Strengthening the Reporting of Observational Studies in Epidemiology (STROBE) tool.

Supplementary Material 3.

## Data Availability

No datasets were generated or analysed during the current study.

## Abbreviations

2D: Two-dimensional
3D: Three-dimensional
6MWD: 6-Minute walk distance
BMI: Body mass index
BODE: Body mass index, airflow obstruction, dyspnea, and exercise
CI: Confidence interval
COPD: Chronic obstructive pulmonary disease
EF: Ejection fraction
FAC: Fractional area change
FEC: Forced expiratory capacity
FEV1: Forced expiratory volume in the first second
FVC: Forced vital capacity
FWS: Free wall strain
GLS: Global longitudinal strain
GOLD: Global Initiative for Chronic Obstructive Lung Disease
LV: Left ventricular
mMRC: Modified medical research council
MPI: Myocardial performance index
PAH: Pulmonary arterial hypertension
PAP: Pulmonary arterial pressure
PASP: Pulmonary artery systolic pressure
PLSS: Peak longitudinal systolic strain
PRISMA: Preferred Reporting Items for Systematic Reviews and Meta-Analyses
PRP: Pulmonary rehabilitation program
PSDD: Peak systolic strain dyssynchrony
PVR: Pulmonary vascular resistance
RV: Right ventricular
SNS: Sympathetic nervous system
STE: Speckle-tracking echocardiography
STROBE: Strengthening the Reporting of Observational Studies in Epidemiology
TAPSE: Tricuspid annular plane systolic excursion
TDI: Tissue Doppler imaging
WU: Wood units
