# Supplementary material for: Speckle-tracking echocardiographic abnormalities in chronic obstructive pulmonary disease: a systematic review and meta-analysis
Source: J Cardiovasc Imaging. 2025 May 8;33:4. doi: 10.1186/s44348-025-00046-5 (PMC12060328; doi:10.1186/s44348-025-00046-5)
Supplement: Supplementary file 3 — Supplementary Material 3. [file 44348_2025_46_MOESM3_ESM.docx]

| Contributor | Concept | Study Design | Data collection | Statistical analysis | Literature overview | Discussion | Fund Generation |
| --- | --- | --- | --- | --- | --- | --- | --- |
| RNV |  | ✓ | ✓ |  |  | ✓ | No fund |
| SKS | ✓ | ✓ | ✓ |  |  | ✓ | No fund |
| ND | ✓ | ✓ |  |  |  | ✓ | No fund |
| MPA |  | ✓ | ✓ |  |  | ✓ | No fund |
| CN |  |  |  | ✓ | ✓ | ✓ | No fund |
| CSH |  |  |  | ✓ | ✓ | ✓ | No fund |
| NP |  |  |  | ✓ | ✓ | ✓ | No fund |
| SAH | ✓ | ✓ |  |  | ✓ | ✓ | No fund |
| CSK | ✓ | ✓ |  |  | ✓ | ✓ | No fund |
